# Predictors, causes, and trends of neonatal mortality at Nekemte Referral Hospital, east Wollega Zone, western Ethiopia (2010–2014). Retrospective cohort study

**DOI:** 10.1371/journal.pone.0221513

**Published:** 2019-10-09

**Authors:** Elias Merdassa Roro, Meseret Itana Tumtu, Dejene Seyoum Gebre

**Affiliations:** 1 Department of Public Health, Institute of Health Sciences, Wollega University, Nekemte, Oromia, Ethiopia; 2 Nekemte Polly Clinic, Nekemte Town, Oromia, Ethiopia; Liverpool School of Tropical Medicine, UNITED KINGDOM

## Abstract

**Background:**

Neonatal mortality is a significant contributor to infant mortality. Causes and predictors of neonatal death are known to vary in different settings and across different contexts. This study aimed to assess predictors, causes, and trends of neonatal mortality amongst neonates admitted to Nekemte Referral Hospital neonatal unit between 2010–2014.

**Methods:**

Retrospective data was collected for 2090 live born neonates admitted to the neonatal intensive care unit of Nekemte Referral Hospital by reviewing records between 2010 to 2014. Variables were collected from the neonatal registration book and patient card on the predictors, causes, and trends of neonatal death using a standard checklist developed by the World Health Organization (WHO). Data was analyzed using Epi info version 3.5.1, and SPSS version 25 for windows. The level of significance was set at P<0.05 with the corresponding confidence intervals at 95%. A logistic regression model was used for analysis and to control for confounders. Microsoft Excel 2007 was used to construct the trend analysis.

**Results:**

There were 183 deaths in the cohort equivalent to 8.8% of deaths among total admitted neonates during the study period. Early neonatal deaths accounted for 8% and late neonatal deaths accounted for 0.71% of deaths among total admitted neonates. Main predictors identified for an increased risk of neonatal mortality were; neonates from rural residents [AOR 1.35, (95% CI, 1.35–1.87)], birth order of greater than five [AOR 5.10, (95% CI, 1.15–22.63)], home delivery [AOR 3.41, (95% CI, 2.24–5.19)], very low birth weight [AOR 6.75, (95% CI, 3.63–12.54)] and low birth weight [AOR 2.81, (95% CI, 1.95–4.05)] and inability to cry at birth [AOR 2.21, (95% CI, 1.51–3.22)]. The trend analysis showed a sharp fall for the neonatal mortality over the last five years with a mean reduction of 16%.

**Conclusions:**

Data from the Nekemte Referral Hospital Neonatal Intensive Care Unit analysis revealed majority of the deaths were occurred during early neonatal period. The main predictors of neonatal mortality identified from this study needs strengthening an appropriate public health intervention through addressing antenatal care, curbing home delivery.

## Introduction

The neonatal period, defined as the first 28 days of life, is the riskiest period for a child’s survival contributing to approximately 44% of under-five deaths and 60% of infant deaths [1,2]. Neonatal mortality is reducing slower than deaths during other periods of childhood [3,4]. Globally, nearly 3 million children die within the neonatal period [2]. Almost all (99%) of these deaths occur in low and middle-income countries, only half of children death within the neonatal period from low- and middle-income countries occurs at health care facilities [1,5].

Neonatal mortalities mainly occur as a result of preventable or treatable causes and conditions such as; prematurity, birth asphyxia, and sepsis, are the three areas that can be mitigated by the presence of a skilled clinicians at the time of delivery [1,6]. Ethiopia has amongst the highest neonatal mortality rates of any country, even when compared to the regional average for Africa [7]. For instance, in 2011, the neonatal mortality rate was 37 per 1000 live births in Ethiopia, compared to 35.9 per 1000 live births in the African region [8]. Despite many efforts from the government and other stakeholders, only a slow decline has been achieved in the last 15 years. For example, the neonatal mortality rate for the years 1991–1995 was 46 per 1000 live birth, reducing to 37 per 1000 live births in the period 2006–2011 [9, 10].

In 2012, Ethiopia introduced the Community-Based Interventions for Newborns in Ethiopia (COMBINE) trial mainly delivered through the Health Extension Package (HEP). It provides a package of community-based interventions in the HEP, which include promotion of antenatal care (ANC), clean and safe delivery, postnatal follow-up of mother and baby, and management of neonatal sepsis by HEWs when referral is not possible. The program is being rapidly scaled up and by the end of 2014 about 102 districts and 2,445 Health Posts were covered. Similarly, initiatives to improve newborn care in health centers and hospitals are ongoing resulting in the establishment of 850 newborn corners in health centers and 30 Neonatal Intensive Care Units in hospitals. During scale-up, the initiative established approximately 30 Neonatal Intensive Care Units in hospitals (NICU) [10]. Nekemte Hospital was one of the health facilities targeted by the program.

In this study, we aimed to identify whether improvements have been observed regarding to Causes, and Trends of Neonatal Mortality since the establishment of the Neonatal Intensive Care Units at Nekemte Referral Hospital over the study period.

## Materials and methods

### The study design, setting and participants

This study was conducted by reviewing a five-year (i.e., during 2010–2014) neonatal registry at the neonatal intensive care unit (NICU) at Nekemte Referral Hospital. Nekemte Referral Hospital is located in East Wollega Zone, Oromia regional estate, western Ethiopia, 331 kilometers from Addis Ababa. It was the only referral hospital in the area during the study period. The hospital has 178 beds and 207 health professionals, consisting of different disciplines, with four inpatient departments, a medical ward, surgical ward, obstetrics and gynecology ward, and pediatrics ward. The Neonatal Intensive Care Unit comprises of 21 beds and is part of the pediatric ward. In this unit, there are five trained nurses and two pediatricians [11]. Trained data collectors collected the data from the standard registration book of NICU from July-September 2015.

Over the study period, (2010–2014), 2110 neonates were admitted to NICU at Nekemte Referral Hospital. Of which, 2090 neonates had complete information relevant to the study and who were included in the study. Twenty of the admitted neonates to the unit had incomplete information for variables like age, sex, and the admission outcomes and, were therefore excluded from the analysis. Data was collected using a data extraction form prepared to extract the necessary information for the study based on the World Health Organization (WHO) standard neonatal register. The data was used to identify the predictors, causes, and trends of neonatal death. To ensure data quality, training was given to all data collectors prior to the start of data collection. The investigators supervised the overall activities of data extraction, and 5% of the data that was collected was randomly selected and checked with the neonatal register by the principal investigators.

### Study variables and definitions

#### Outcome variable

Neonatal admission outcome was the dependent variable, categorized as neonatal death and survivors. Any neonate admitted to Nekemte Referral Hospital NICU who died within the unit during the neonatal period, (within the first 28 days after live birth) was categorized as ‘neonatal death/died.’ Neonates admitted to Nekemte Referral Hospital NICU who survived the first 28 days after live birth were categorized as survivors.

#### Independent variables

Factors included in the analysis baseline factors: age of the mother at child birth in presented full years, marital status measured as currently in marriage and currently not in marriage, religion, and residence categorized as rural and urban. Pregnancy and related delivery variables such as; gestational age (calculated from the last menstrual period), antenatal care visits categorized as whether the mother had antenatal care visits despite the number of visits and contents antenatal care visits for the current child, place of delivery represents where the current child born and categorized as home or health institution, mode of delivery for the current child and categorized as whether the child was borne by Caesarean section, vaginal delivery (as spontaneous or Assisted), history of neonatal death refers to whether the mother encountered neonatal deaths throughout her marriage history, birth weight refers measured at birth if the child was born in health institutions or estimated if the child was born at home, birth order refers to the total number births a mother have including the current child and ability to cry refers to the ability a child to cry (makes sound) at birth.

### Statistical methods

The raw data used for this study was obtained from the neonatal register over the years 2010–2014. Data was entered and cleaned using Epi Info version 3.5.1 and exported to SPSS version 24 statistical software for Windows for analysis. Data were checked for normality assumption and results were presented in tables, graphs and pie charts as necessary. Multicollinearity test was performed and those variables showed collinear were excluded from analysis. Univariate analysis was performed to screen predictors of neonatal mortality for each variable one at a time chi-square tests were conducted and p-values were reported. At bivariable level, Crude Odds Ration at 95% with Confidence Intervals were reported. Then, those variables with a P-value<0.05 and other with biological importance at bivariable analysis were collected and entered into multiple logistic regression to control for possible confounders. As Binary Logistic Regression (BLR) was the model used for multivariable analysis, it was used in this study to compare two groups, i.e. ‘neonatal death’ and ‘survivors of the neonatal period.’ It was also preferable because the dependent variable had binary outcomes. Effect sizes were presented using crude and adjusted odds ratio at 95% CI. In this section, we followed a similar approach from a published paper and modified to this study [12]. Neonatal mortality was set as the dependent variable and tested for association with socio-demographic and maternal/newborn factors. The final mortality trend curve was created using Microsoft Excel 2007.

### Ethics approval and consent to participate

This study was approved by the Wollega University institute of Health Sciences Research Ethics Committee. A letter of permission was also obtained from Nekemte Town Health Office to conduct the study. Data was collected after getting official permission from Nekemte Referral Hospital administration office. Individual identifiers were removed to maintain the anonymity of patients by assigning a unique number to each questionnaire. All data was collected from the register which was kept in a secure place and all data were fully anonymized before we accessed them. After collection of the data, all the patient records and patients’ cards were placed back into a secure place. Data were not be shared with anybody other than authors for ethical reasons. All data were entered into password protected computer. Only the investigators had access to the data. The ethics committee waived the requirement for informed consent.

## Results, discussion and conclusion

### Results

#### Socio-demographic profile of the study population

During the study period, (2010–2014), 2110 neonates were admitted to NICU at Nekemte Referral Hospital. From these neonates admitted, 20 (0.95%) had incomplete information regarding their age, sex or admission outcome in the registration book and were excluded from the analysis. The data presented in this study was collected and analyzed for the remaining 2090 admitted neonates to the unit who had complete information (variables). Most, 1840 (96.49%), of the mothers in the neonates that survived were aged 18–34 years. The mothers of 90.71% of the neonates that died were also aged 18–34. Almost all, 180 (98.36%) of mothers whose neonate died, and 1894 (99.32%) of mothers whose neonate survived were currently married. A similar proportion, 97 (53.0%) of the mothers whose neonate died and 983 (51.5%) of those whose neonate survived were Orthodox Christians. 120 (65.57%) mothers whose neonate died were rural residents, compared to1092 (57.26%) mothers whose neonate survived (Table 1).

**Table 1:** Socio-demographic characteristics of mothers of neonates admitted to Nekemte Referral Hospital Nicu east Wollega Zone, Oromia region from 2010–2014.

Characteristics (Variables)	Admission outcomes		p-value
	Died (n = 183)	Survived (n = 1907)	
**Age of the mother at childbirth**			
<18yrs	3 (1.60%)	18 (0.94%)	**0.001**
18–34 yrs	166 (90.71%)	1840 (96.49%)	
>35yrs	14 (7.70%)	49 (2.57%)	
**Residence**			
Urban	63 (34.43%)	815 (43.74%)	**0.049**
Rural	120 (65.57%)	1092 (57.26%)	
**Marital status**			
Not in marriage	3 (1.60%)	13 (0.68%)	0.138
Married	180 (98.36%)	1894 (99.32%)	
**Religion**			
Orthodox Christian	97 (53.0%)	983 (51.5%)	0.394
Protestant Christian	57 (31.1%)	662 (34.7%)	
Muslim	29 (15.8%)	262 (13.7%)	

#### Pregnancy and delivery-related characteristics

A higher number, 1510 (79.2%), of mothers whose neonate survived attended antenatal care compared to the mothers of neonates that died, 107 (58.5%). Relatively higher proportion neonates that died were preterm, 28 (15.3%), relative to 178 (9.3%) in those that survived. Most of the neonates that survived, 1739 (91.2%), were born in a health institution compared to 128 (69.9%) of those that died (Table 2).

**Table 2:** Pregnancy and delivery related characteristics of study participants in Nekemte Referral Hospital Nicu east Wollega Zone, Oromia region 2010–2014.

Characteristics (Variables)	Neonates admission out-comes		p-value
	Died (n = 183)	Survived (n = 1907)	
**Antenatal Care visits**			
Yes	107 (58.5%)	1510 (79.2%)	**0.000**
No	76 (41.5%)	397 (20.8%)	
**Gestational age at delivery**			
Preterm	28 (15.3%)	178 (9.3%)	0.076
Term	153 (83.6%)	1710 (89.7%)	
Post term	2 (1.1%)	19 (1.0%)	
**Mode of delivery**			
Spontaneous vaginal Delivery	131 (71.6%)	1231 (64.6%)	0.065
Caesarean section	25 (13.7%)	390 (20.5%)	
Assisted vaginal Delivery	27 (14.8%)	286 (15.0%)	
**Place of delivery**			
Health facility	128 (69.9%)	1739 (91.2%)	**0.000**
Home	55 (30.1%)	169 (8.8%)	
**Maternal HIV status**			
Positive	1 (0.56%)	14 (0.7%)	**0.004**
Negative	147 (80.3%)	1661 (87.1%)	
Unknown	36 (19.7%)	232 (12.2%)	

#### Delivery related characteristics

Of the total admitted neonates, 1810 (94.91%) of the survived, and 168 (91.80%) died neonates were aged 0–6 days. Twenty-three (12.57%) that died had a very low birth weight < 1500grm compared to 59 (3.10%) that survived the neonatal period. There were more males in both the neonatal deaths (66.12%) and those who survived (64.71%). Hundred thirty-six (74.32%) of neonates that died cried at birth compared to 1606 (84.22%) of those that survived. Only 122 (7.3%) of neonates that died were resuscitated at birth compared to 1545 (92.7%) of survivors of the neonatal period (Table 3).

**Table 3:** Delivery related characteristics of neonates admitted to Nekemte Referral Hospital Nicu east Wollega Zone, Oromia region 2010–2014.

Characteristics (variables)	Neonates admission outcomes		p-value
	Died (n = 183)	Survived (n = 1907)	
**Age of newborn**			
7–28 days	15 (8.20%)	97 (5.10%)	0.054
0–6 days	168 (91.80%)	1810 (94.91%)	
**Birth weight in gram**			
< 1500grm	23 (12.57%)	59 (3.10%)	**0.000**
1500-2499grm	80 (43.72%)	469 (24.59%)	
2500-4000grm	80 (43.72%)	1379 (72.31%)	
**Sex of newborn**			
Male	121 (66.12%)	1234 (64.71%)	0.485
Female	62 (33.88%)	673 (35.29%)	
**Birth order**			
One	66 (36.70%)	971 (50.92%)	**0.000**
Two	7 (3.82%)	60 (3.14%)	
Three	38 (20.77%)	347 (18.20%	
Four	33 (18.03%)	329 (17.25%)	
Five & above	39 (21.31%)	200 (10.50%)	
**Newborn cried at birth**			
Yes	136 (74.32%)	1606 (84.22%)	**0.000**
No	47 (25.68%)	301 (15.78%)	
**Newborn resuscitation at birth**			
Yes	122 (7.3%)	1545 (92.7%)	**0.000**
No	61 (14.4%)	362 (85.6%)	
**Breastfeeding initiation time**			
<1hr	69 (37.7%)	1582 (83.0%)	**0.000**
>1hr	114 (62.3%)	325 (17.0%)	

#### The magnitude of neonatal mortality

There were 183 deaths equivalent to a mean neonatal mortality rate during the study period of 8.8%, (95% CI,7.6–10.1). Early neonatal mortality (defined as death between days 0–6) rate was 8.0%, (95% CI, 93.6–95.5) and while late neonatal mortality rate (defined as death between days 7–28) was 0.7%, (95% CI,4.5–6.4). Though the number of admissions to the unit showed increments over the study period, a sharp decline in neonatal mortality was observed (Table 4).

**Table 4:** Admissions and mortality indices of neonates admitted to Nekemte Referral Hospital Nicu, east Wollega Zone, Oromia region 2010–2014.

Year of admissions	The total number of admissions per year.	A total number of neonatal deaths per year.	Total number of early death (0–6 days) per year.	Total number of late neonatal deaths (7–28 days) per year.	p-value
2010	101	27 (26.7%)	24 (23.7%)	3 (3%)	**0.000**
2011	103	23 (22.3%)	19 (18.4%)	4 (3.9%)	
2012	96	16 (16.6%)	15 (15.6%)	1 (1.4%)	
2013	705	54 (7.7%)	51 (7.25%)	3 (0.43%)	
2014	1085	63 (5.8%)	59 (5.4%)	4 (0.37%)	
Total	2090	183 (8.8%)	168 (8.0%)	15 (0.7%)	

#### Causes of neonatal mortality

**Fig 1:**
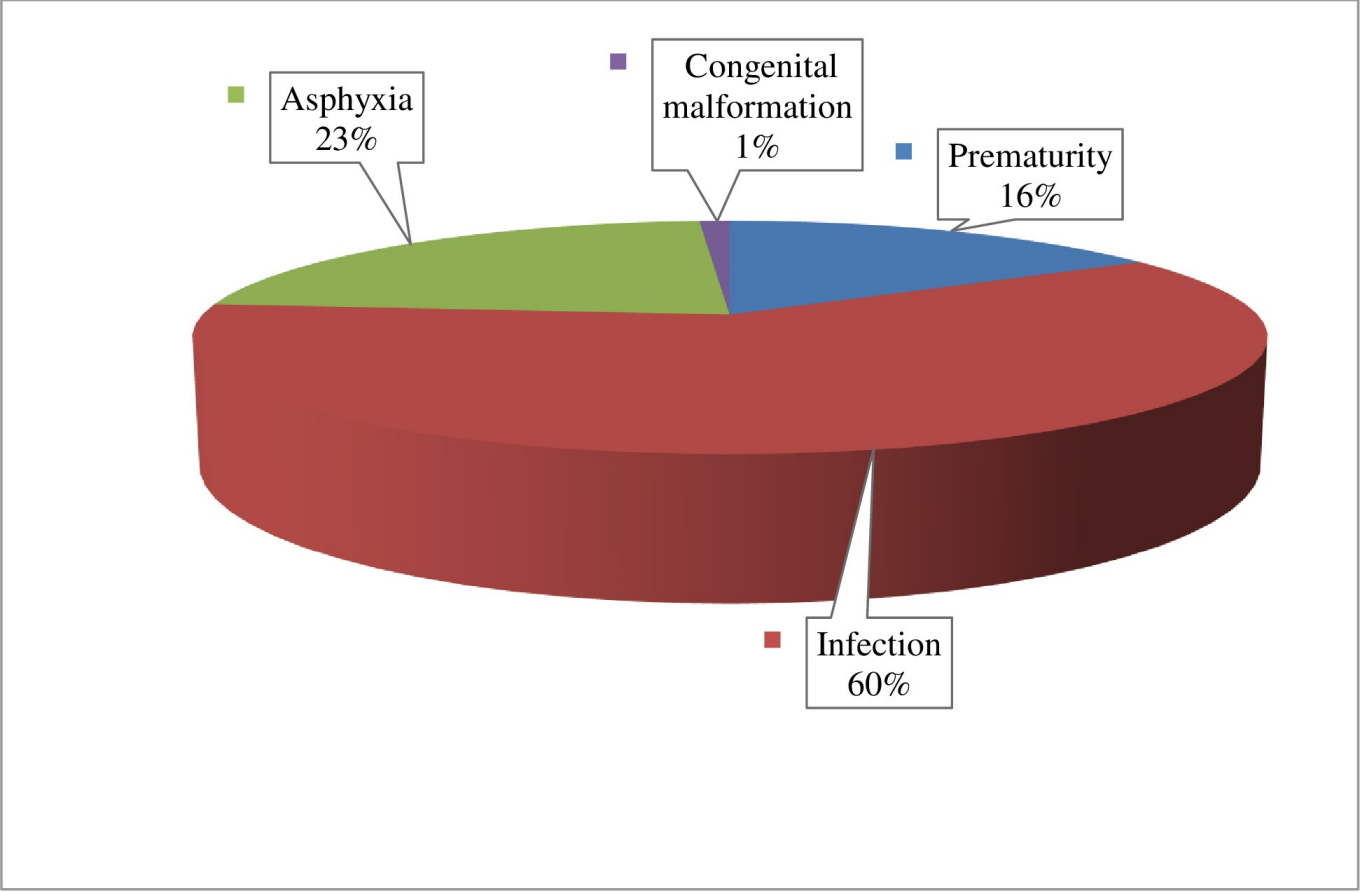
Causes of neonatal mortality among admitted neonates to Nekemte Referral Hospital Nicu of 2010–2014.

The causes of mortality identified at admission to NICU were sepsis (47.8%), birth asphyxia (18%) and premature birth (12.2%). Ninety percent of admitted neonates were treated with an antibiotic, 60% were treated with glucose and 24% were treated with oxygen. The major causes of neonatal death during the study period were; infection (60%), asphyxia (23%), and premature birth 16%) (Fig 1).

#### Socio-demographic predictors of neonatal mortality

After adjusting for socio-demographic factors, rural dwellers were at 1.4 times higher risk of facing neonatal death [AOR 1.35, (95% CI, 1.35–1.87)] during the neonatal period compared to neonates born to urban residents. On the contrary, mothers aged 18–34 years was found to be protective for neonatal death [AOR 0.35, (95% CI, 0.19–0.64)] compared to neonates born to mothers older than 35 years (Table 5).

**Table 5:** Socio-demographic factors associated with neonatal mortality admitted to Nekemte Referral Hospital Nicu, east Wollega Zone, Oromia region from 2010–2014.

Characteristics (Variables)	Neonates admission outcomes		COR 95%CI	AOR 95% CI
	Died (n = 183)	Survived (n = 1907)		
**Age of the mother at childbirth**				
<18yrs	3 (1.60%)	18 (0.94%)	0.583 (0.150–2.270)	0.470 (0.105–2.110)
18-34yrs	166 (90.71%)	1840 (96.49%)	**0.316 (0.171-.584)**	**0.345 (0.185–0.643)**
>35yrs	14 (7.70%)	49 (2.57%)	1	1
**Residence**				
Urban	63 (34.43%)	815 (43.74%)	1	1
Rural	120 (65.57%)	1092 (57.26%)	**1.422 (1.034–1.954)**	**1.350 (1.350–1.867)**
**Marital status**				
Not in marriage	3 (1.60%)	13 (0.68%)	2.428 (0.686–8.600)	1.867 (0.117–2.021)
Married	180 (98.36%)	1894 (99.32%)	1	1
**Religion**				
Orthodox Christian	97 (53.0%)	983 (51.5%)	1	1
Protestant Christian	57 (31.1%)	662 (34.7%)	0.873 (0.620–1.228)	0.937 (0.662–1.324)
Muslim	29 (15.8%)	262 (13.7%)	1.122 (0.725–1.736)	1.057 (0.679–1.647)

#### Delivery and pregnancy outcome related predictors of neonatal mortality

After adjusting for delivery and pregnancy related outcomes, potential predictors for neonatal mortality were; home delivery [AOR 3.41, (95% CI, 2.24–5.19)], very low birth weight (<1500g) [AOR 6.75, (95% CI,3.63–12.54)], low birth weight (1500-2499g) [AOR 2.81, (95% CI,1.95–4.05)], birth order of greater than five [AOR 5.103, (95% CI, 1.15–22.63)] and inability to cry at birth [AOR 2.205, (95% CI, 1.51–3.22)] strongly predicted an increased risk of neonatal death (Table 6).

**Table 6:** Delivery and pregnancy outcome related factors associated with neonatal mortality in the Nicu of Nekemte Referral Hospital in the east Wollega Zone, Oromia region from 2010–2014.

Characteristics (Variables)	Neonates admission outcomes			
	Died (n = 183)	Survived (n = 1907)	COR 95%CI	AOR 95%CI
**Mode of delivery**				
Caesarean section	25 (13.66%)	390 (20.45%)	1	1
Spontaneous vaginal delivery	131 (71.6%)	1231 (64.6%)	**0.577 (0.368–0.904)**	1.165 (0.717–1.892)
Assisted vaginal delivery	27 (14.8%)	286 (15.0%)	0.93 (0.602–1.416)	1.434 (0.894–2.300)
**Place of delivery**				
Health facility	128 (69.9%)	1739 (91.2%)	1	1
Home	55 (30.1%)	169 (8.8%)	**4.594 (3.230–6.534**)	**3.412 (2.242–5.194)**
**Gestational age**				
Term	153 (83.61%)	1710 (98.67%)	1	1
Pre-term	28 (15.30%)	178 (9.33%)	**1.758 (1.142–2.707)**	1.121 (0.674–1.867)
Post term	2 (1.09%)	19 (1.00%)	1.176 (0.271–5.098)	1.059 (0.211–5.315)
**Gravida**				
One	65 (35.52%)	954 (50.03%)	1	1
Two to three	82 (44.81%)	726 (38.07%)	**1.658 (1.181–2.238)**	1.237 (0.438–3.489)
≥Four	36 (19.67%)	227 (11.90%)	**2.238 (1.511–3.586)**	0.566 (0.148–2.169)
**Number of live births**				
One	73 (39.89%)	1023 (53.64%)	1	1
Two to three	75 (40.98%)	657 (34.45%)	**1.600 (1.142–2.240)**	0.583 (0.182–1.873)
≥Four	35 (19.13%)	227 (11.90%)	**2.161 (1.409–3.314)**	0.054 (0.002–1.177)
**Birth order**				
One	66 (36.70%)	971 (50.92%)	1	1
Two	7 (3.82%)	60 (3.14%)	2.182 (0.941–5.060)	1.255 (0.375–4.203)
Three	38 (20.77%)	347 (18.20%	1.381 (0.795–2.398)	1.793 (0.516–6.230)
Four	33 (18.03%)	329 (17.25%)	1.376(0.656–2.885)	1.653 (0.464–5.896)
Five & above	39 (21.31%)	200 (10.50%)	**1.990 (1.063–3.726)**	**5.103 (1.151–22.629)**
**Age of newborn**				
7–28 days	15 (8.20%)	97 (5.10%)	1	1
0–6 days	168 (91.80%)	1810 (94.91%)	0.607 (0.345–1.069)	1.170 (0.631–2.171)
**Birth weight in gram**				
<1500g	23 (12.57%)	59 (3.10%)	**7.013 (4.109-11-969)**	**6.745 (3.630–12.535)**
1500-2499g	80 (43.72%)	469 (24.59%)	**2.888 (2.082–4.006)**	**2.812 (1.951–4.053)**
2500-4000g	80 (43.72%)	1379 (72.31%)	1	1
**Sex of newborn**				
Male	121 (66.12%)	1234 (64.71%)	1	1
Female	62 (33.88%)	673 (35.29%)	0.94 (0.682–1.294)	0.911 (0.651–1.277)
**Cried at birth**				
Yes	136 (74.32%)	1606 (84.22%)	1	1
No	47 (25.68%)	301 (15.78%)	**1.844 (1.295–2.626)**	**2.205 (1.507–3.224)**

#### Trend analysis

**Fig 2:**
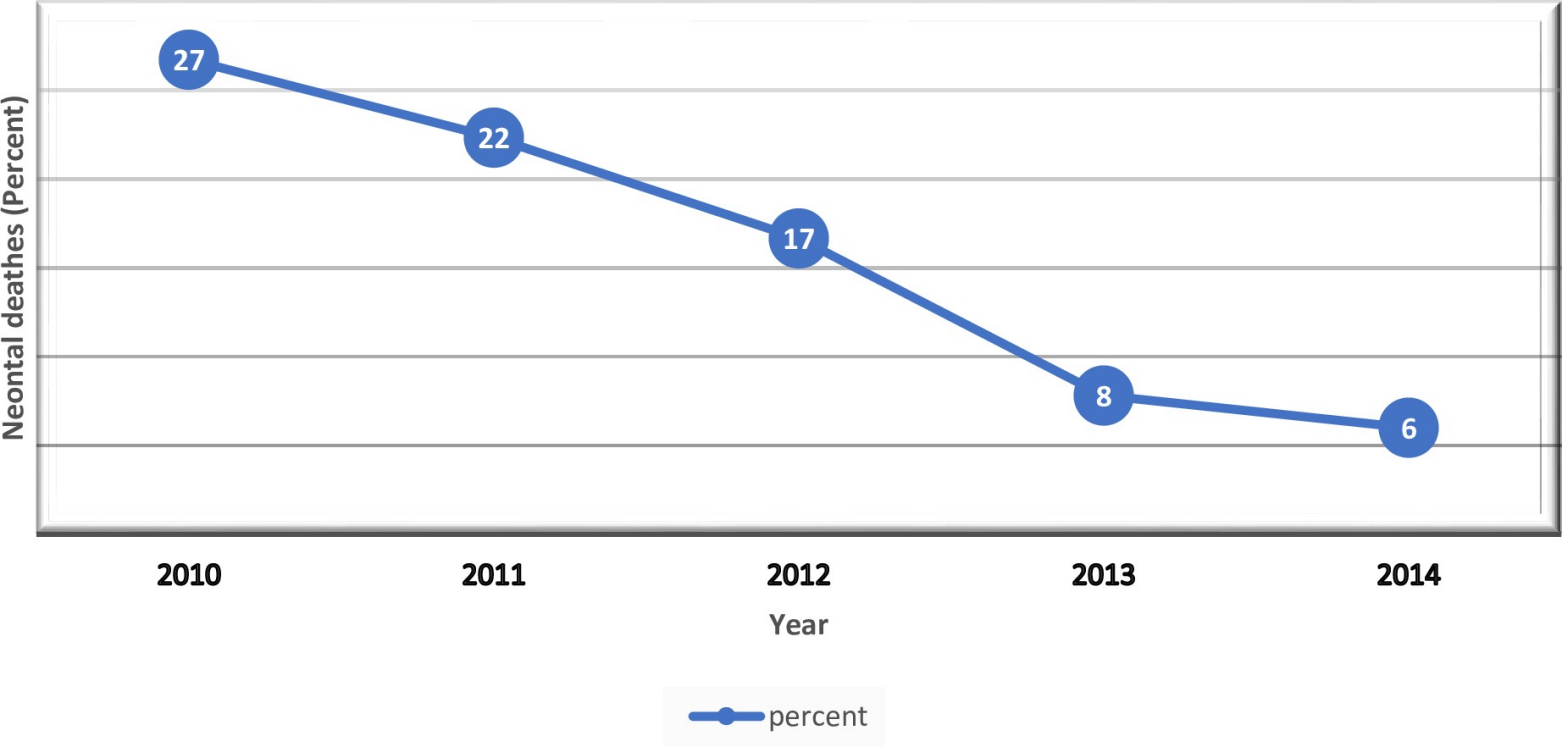
Trends of neonatal mortality by year in Nekemte Hospital Nicu, east Wollega Oromia 2010–2014.

Over the five-year analysis (2010–2014), the trends of neonatal mortality showed a declining pattern. As the figure below shows, the number of neonatal deaths was 27% in 2010 and 7% in 2014, with a mean decrement of neonatal death of 16% P<0.000 (Fig 2).

### Discussion

In this study facility-based cohort study, the finding reported that neonatal mortality was declining during the five years of study period. After adjusting for confounders, potential predictors of neonatal mortality identified were; birth order of greater than five, home delivery, birth weights and inability to cry at birth.

#### The magnitude of neonatal mortality

Our analysis reported magnitude of a neonatal mortality of 8.8% of the total neonates admitted to Nekemte Referral Hospital NICU during the period 2010–2014. This finding is lower than the study conducted in Tikur Anbesa Specialized Hospital NICU in Ethiopia which reported neonatal mortality of 23.3% [13], and study in Jimma zone, Ethiopia reported 35.5% NMR [14], A community-based study in urban Pakistan which reported a 49% neonatal mortality [15]. This finding also revealed that the majority, 92%, of the deaths were in the first week. This could be due to the majority of neonatal deaths in low- and middle-income countries being related to labour, intrapartum and immediate newborn care practices.

Though the number of admissions increased during the study period, our analysis showed a decline in neonatal mortality. This declining trend was also observed in both early and late neonatal mortalities which could be attributed to the expansion of the NICU, improvements of facilities and training of health professionals that might have aided these better outcomes. For instance, the NICU was part of the main hospital until 2012, but later shifted to a newly constructed independent unit. This new unit is clean, well ventilated and better equipped. It should be noted here that this decline in NMR is still slow compared to declines in under-five mortality in Ethiopia.

#### Causes of neonatal deaths

Most of the causes of neonatal death reported in this study were infection, prematurity, and asphyxia. This finding is comparable to other studies [1, 2, 3,5, 15,16]. These conditions are readily preventable. This study indicated a high early neonatal mortality, especially within the first 24 hours of birth, suggesting that neonatal survival interventions have to target the intrapartum as well as immediate and early neonatal periods. One systematic review reported that care during labour and birth could help reduce intrapartum deaths by 25% - 85% [7]. However, access to such services is difficult, especially in rural areas where transport to the specialist care centers is not easily available [17].

One study revealed that at birth, about 5% - 10% of newborns required resuscitation. For this, training on appropriate resuscitation techniques was reported to reduce deaths in babies with intrapartum asphyxia by 30% and early neonatal deaths by 38% [18]. These are major causes of neonatal mortality in this study.

#### Predictors of neonatal mortality

In multivariate analysis, neonatal mortality was associated with; home delivery, birth order of greater than five, low birth weight, inability to cry at birth and being a rural dweller.

#### Residence and neonatal mortality

In this study, rural dwellers experienced higher neonatal mortality than their urban counterparts. The main reason may be related to a low level of maternal health service utilization amongst rural women compared with their urban counterparts. As in most sub-Saharan countries, urban women in Ethiopia tend to benefit from better awareness and access to services than rural mothers. In addition, health institutions are typically concentrated in urban areas. Moreover, previous studies have found that rural women are more readily influenced by traditional practices that are contrary to modern health care [19]. These elements potentially explain the close connection between residence, use of maternal health services and ultimately NMR.

#### Place of delivery and neonatal mortality

Neonates born at home had increased risk of death during the neonatal period compared to those who gave birth in health facilities. Women who gave birth at home experienced 3.4 times higher odds of neonatal death than their counterparts. A similar finding was reported in pooled studies in low and middle-income countries (LMIC) [20]. Though home delivery had a high risk of neonatal deaths, recent findings from Ethiopia reported most women had a strong aspiration to give birth at home considering it a natural space for delivery and allowing for traditional events related to births to take place and thus making it more enjoyable. Even those who gave birth in health institutions appreciated events in connection to home delivery [21]. Among the many reasons that discouraged facility delivery, 80% of respondents believed that a lack of respectful care discouraged women from having a facility-based birth [22]. Hence instilling a system-wide culture of respect in health facilities, that will protect the rights of both women and service providers could be one solution.

#### Birth weights and neonatal mortality

Low weight for birth was another predictor of neonatal mortality [23,24,25], a fact that has been confirmed in many studies conducted in Ethiopia and Brazil, which found that those born alive, but with low weight for birth were at higher risk of death than those with a normal birth weight in the first month of life.

In this study, all infants born with a birth weight of less than 2500g were categorized as ‘low for birth weight’, the likelihood of neonatal death increased with decreasing birth weight [23,26], however definitions of low birth weight (LBW) vary across these studies and results should be interpreted with caution [12]. To reduce the adverse effects of LBW on newborns, appropriate care of LBW infants, including their feeding, temperature maintenance, hygienic cord, and skin care, and early detection and treatment of infections and complications including respiratory distress syndrome can substantially reduce mortality [27].

#### Trend and causes of deaths

In this study, there was a sharp decline in neonatal mortality with a mean reduction of 16% from 2010–2014, but the trend was stagnant at 3.2% per annum p<0.000. A similar decline has been reported from other LMICs at 17% [17], compared to the global NMR reduction of 28% during the same period as our study [21]. Greater reductions in NMR have been seen in in North Africa, compared to sub-Saharan Africa [17], but all regions have shown steady reductions.

The stagnation in decline of NMR may be due to non-preventable causes at the NICU level such as prematurity and congenital abnormality, which require prevention at the public health level such as maternal malnutrition, prevention of malaria and anemia. In this study, Perinatal asphyxia did not show any change and may be due to delays in accessing appropriate care. Infection reduced to nearly zero in the five-year study period, however deaths related to prematurity and asphyxia were stagnant.

## Limitation of this study

Due to the retrospective nature of the data used in this study and the incompleteness of the register, some missing variables such as maternal education, birth interval and income of the mother were not analyzed but might be important predictors of neonatal mortality. Our study inferred to study set up during the study period. We are unable to get a well-organized baseline data before the start of the programs to compare with the current data to assess to effectiveness of the program.

## Conclusions

The 2010–2014 Nekemte Hospital NICU data demonstrated significant changes in neonatal mortality trend. Being a rural resident, home delivery, high birth order, low & very low birth weight and inability to cry at birth were found to be main predictors of neonatal mortality. These findings call for appropriate antenatal care, particularly in high risk groups who can be distinguished and managed. Promotion of institutional delivery has to be strengthened. We believe that the findings of this study will provide substantial evidence for the care providers; program implementers and policymakers and help them to pass evidence-based decisions and take timely interventions within the study setup.

## Abbreviations

WHO: World Health Organization
CI: Confidence Interval
AOR: Adjusted Odds Ratio
OR: Odds Ratio
EDHS: Ethiopian Demographic and Health Survey
VA: Verbal Autopsy
CODA: Cause of Death Assignment
SD: Standard Deviation
ANC: Ante-Natal Care
